# Safety and efficacy of the traditional Chinese medicine chaihu guizhi ganjiang decoction combined with wendan decoction in the treatment of premature ejaculation: an open-label retrospective cohort study on Chinese men

**DOI:** 10.1093/sexmed/qfag031

**Published:** 2026-05-09

**Authors:** Sijia Sun, Zhou Zhao, Shuyi Huang, Li Ling, Dawei Gao, Wenxiu Zhang, Baofang Jin, Dalin Sun

**Affiliations:** School of Medicine, Southeast University, Nanjing 210009, PR China; School of Medicine, Southeast University, Nanjing 210009, PR China; School of Medicine, Southeast University, Nanjing 210009, PR China; Reproductive Center, Zhongda Hospital, Southeast University, Nanjing 210009, China; Faculty of Chinese Medicine, Macau University of Science and Technology, Macau 999078, China; Department of Traditional Chinese Medicine, Qinghai University Medical College, Xining 810001, China; Andrology Department of Integrative Medicine, Zhongda Hospital, Southeast University, Nanjing 210009, China; Andrology Department of Integrative Medicine, Zhongda Hospital, Southeast University, Nanjing 210009, China

**Keywords:** premature ejaculation, Chaihu Guizhi Ganjiang Decoction, Wendan Decoction, Dapoxetine

## Abstract

**Background:**

Chaihu Guizhi Ganjiang Decoction combined with Wendan Decoction (CGGWD) is used clinically to treat liver depression and spleen deficiency-related premature ejaculation (PE), while clinical evidence and safety are still limited.

**Aim:**

To assess the safety and efficacy of the Traditional Chinese Medicine (TCM) formula, CGGWD, in comparison with dapoxetine for PE.

**Methods:**

This retrospective study included male PE patients with liver depression and spleen deficiency treated at a prominent academic medical center (November 2021-March 2025). Patients completed a 4-week regimen, with sexual function and systemic symptoms evaluated before and after treatment.

**Outcomes:**

Outcomes were determined based on assessment of intravaginal ejaculatory latency time (IELT), Premature Ejaculation Diagnostic Tool (PEDT), Traditional Chinese Medicine Quality of Life Evaluation Scale (CQ-11D), Global Rating of Sexual Satisfaction (GRISS), and Clinical General Impression of Change (CGIC).

**Results:**

A total of 226 patients were enrolled in the study, including 165 in the CGGWD group and 61 in the dapoxetine group. After 4 weeks of treatment, the dapoxetine group showed an increase in IELT from 1.5 to 3.0 min, a decrease in PEDT scores from 13 to 9, and a reduction in GRISS scores from 37 to 28 (all *P* < .05), with a CGIC score of 2. In the CGGWD group, IELT increased from 1.5 to 3.5 min, PEDT scores decreased from 12 to 9.5, and GRISS scores dropped from 36 to 26.5 (all *P* < .05), also yielding a CGIC score of 2. No significant differences were observed between the 2 groups regarding changes in IELT, PEDT, GRISS, or overall clinical impression (*P* = .978, .567, .883, and .993, respectively). However, regarding the CQ-11D score, the dapoxetine group remained stable at 24 points, whereas the CGGWD group decreased significantly from 24 to 19 points (*P* < .05). Furthermore, the incidence of adverse events was significantly lower in the CGGWD group compared to the dapoxetine group (7 cases vs. 29 cases, *P* < .05).

**Clinical Implications:**

The TCM formulation, CGGWD, demonstrates both safety and efficacy in the management of PE.

**Strengths and Limitations:**

This study offers the initial clinical validation of CGGWD for PE but is limited by population selection constraints, potential information bias, and unobserved confounding factors.

**Conclusion:**

For Chinese men with liver depression and spleen deficiency PE, CGGWD matches dapoxetine in efficacy but offers superior safety and systemic symptom relief, serving as a safe, effective alternative.

## Introduction

Premature ejaculation (PE) is a prevalent male sexual dysfunction that affects more than 20% of men globally. In Europe and America, the prevalence of PE is approximately 22.7%,^1^ while higher rates have been reported in Asia and Latin America.^2^ A study conducted by McMahon et al. in the Asia-Pacific region revealed that up to 31% of men experienced PE or suspected PE; however, epidemiological evidence suggests that the actual prevalence of PE may be underestimated in diagnostic rates, potentially due to factors such as social stigma.^3^^,^^4^

Research indicates that, in comparison to men without PE, individuals with PE are more susceptible to concurrent sexual dysfunctions (including orgasmic disorders, low libido, and erectile dysfunction) as well as psychological issues such as depression and anxiety. These conditions significantly impact the quality of life, psychosocial health, and relational dynamics of both the patients and their partners.^5^

Consequently, addressing the diagnosis and treatment of PE holds substantial social significance. Despite increasing access to information, many men remain undiagnosed or untreated due to psychological and societal barriers, opting instead for traditional remedies.^6^

Currently, standard treatments for PE include behavioral therapy, topical anesthetics, dapoxetine, and other selective serotonin reuptake inhibitors (SSRIs). Most pharmacological interventions target neurotransmitters involved in the ejaculatory process, such as serotonin and oxytocin. However, these treatments typically yield only modest effects, providing temporary prolongation of the ejaculatory latency period, with a high likelihood of recurrence upon cessation.^7^ Additionally, they may induce systemic side effects, such as nausea, dizziness, somnolence, headache, diarrhea, and insomnia associated with dapoxetine use.^8^ Thus, identifying novel, safe, and effective therapeutic options remains a critical priority in clinical practice.

Chinese herbal medicine, an integral component of Traditional Chinese Medicine (TCM), has garnered increasing attention in recent years for its potential application in PE treatment,^9^ although robust evidence-based support remains limited. In the context of TCM, the etiology and pathogenesis of PE are primarily associated with various syndrome types, including Yin deficiency with hyperactivity of fire, kidney deficiency with instability, damp-heat pouring downward,^10^ etc. Among these, liver depression and spleen deficiency are recognized as the most prevalent clinical pattern. Chaihu Guizhi Ganjiang Decoction combined with Wendan Decoction (CGGWD) is a classic TCM prescription specifically indicated for regulating this syndrome.

Our research team has previously obtained preliminary favorable clinical outcomes in the management of patients with liver depression and spleen deficiency pattern of PE using CGGWD. Despite these preliminary clinical observations, the definitive efficacy and safety profile of CGGWD for liver depression and spleen deficiency pattern PE have not yet been systematically elucidated, and real-world comparative data of CGGWD versus dapoxetine, the first-line pharmacotherapy for PE, in this specific TCM syndrome population remain scarce. Therefore, in this retrospective, open-label study, we sought to evaluate the safety and efficacy of CGGWD in comparison with dapoxetine for the treatment of liver depression and spleen deficiency pattern PE in a real-world clinical setting, and address the following 2 core questions: does CGGWD significantly improve ejaculation function after 4 weeks of treatment in Chinese men with liver depression and spleen deficiency pattern of PE? What is the incidence of treatment-related adverse events of CGGWD in this target population?

## Methods

### Study design

This investigation was conducted as a retrospective cohort study, encompassing a total of 226 male patients diagnosed with PE and conforming to the TCM syndrome classification of liver depression and spleen deficiency. The study was carried out at a tertiary university-affiliated hospital, from November 2021 to March 2025. This study received review and approval from the Clinical Research Ethics Committee of the participating institution. Informed written consent was obtained from all participants prior to their inclusion in the study.

### Definition

The diagnostic criteria for PE in Western medicine were based on the guidelines provided by the International Society for Sexual Medicine (ISSM). The criteria for diagnosing PE include: (1) ejaculation that always or nearly always occurs prior to or within about 1 min of vaginal penetration from the first sexual experience (lifelong PE) OR a clinically significant reduction in IELT, often to about 3 min or less (acquired PE); (2) an inability to delay ejaculation during all or nearly all vaginal penetrations; and (3) the presence of negative personal consequences, such as distress, anxiety, or depression.^11^

The criteria for syndrome differentiation in TCM are based on the “Guiding Principles for Clinical Research of New TCM Drugs (Trial)” issued by the National Medical Products Administration.^12^ In TCM, the syndrome of liver depression and spleen deficiency is characterized by a constellation of symptoms arising from the liver's inability to regulate the flow of *qi* and the spleen’s impaired capacity to transport *qi*. The primary clinical manifestations include distension and pain in the epigastric or hypochondriac regions, abdominal distension, poor appetite, and loose stools. Secondary symptoms may encompass psychological disturbances such as depression or irritability, frequent sighing, intestinal rumbling, flatulence, abdominal pain that precedes diarrhea, relief of pain following diarrhea, a white or greasy tongue coating, and a taut or fine pulse. A diagnosis of liver depression and spleen deficiency can be established if 3 primary symptoms are observed, or if 2 primary symptoms and 2 secondary symptoms are present.

### Inclusion and exclusion criteria

The inclusion criteria for participants in this study are as follows: (1) Individuals with primary or secondary PE that fulfills the diagnostic criteria for PE; (2) Age range of 18 to 69 years; (3) A history of PE persisting for more than 6 months; (4) A stable sexual partnership with a frequency of sexual activity of at least 4 times per month for a minimum duration of 6 months; (5) Normal erectile function as determined by a score greater than 21 on the 5-item version of the International Index of Erectile Function (IIEF-5); (6) Conformance with the TCM syndromes diagnostic criteria for liver depression and spleen deficiency type. The exclusion criteria are as follows: (1) Presence of organic lesions in the urinary tract, including but not limited to neurogenic bladder, urethral stricture, prostate cancer, urinary tract infection, tuberculosis, calculi, and other related conditions; (2) Conditions such as epididymitis, varicocele, lumbar intervertebral disc protrusion, and other similar diseases; (3) Severe mental and psychological disorders, allergic conditions, and liver or kidney dysfunction; (4) Allergy to the medications utilized in this study; (5) Participation in other clinical trials within the preceding 3 months; (6) Patients who were lost to follow-up or whose data were incomplete during the treatment period.

### Participant allocation

Participants were allocated to either the treatment group or the control group according to their individual preferences and the clinical judgment of their attending physicians. This method of grouping was selected to more accurately mirror real-world clinical practice and to enhance patient compliance and meet ethical standards.

### Treatment methodology

Participants were exclusively included in one of 2 monotherapy regimens: the dapoxetine group and the CGGWD group. To maintain the integrity of the monotherapy design, individuals in the dapoxetine group were explicitly instructed to abstain from using any Chinese herbal medicines throughout the study duration. Conversely, participants in the CGGWD group were prohibited from using dapoxetine or any other PDE5 inhibitors. All dosages were prescribed by clinicians in accordance with established dosing guidelines.

The formulation and dosage of CGGWD are specified as follows: *Bupleurum chinense* DC. 12 g, *Scutellaria baicalensis* Georgi 10 g, *Cinnamomum cassia* (L.) J.Presl 10 g, *Zingiber officinale* Rosc. 15 g, *Ostrea gigas* Thunb. ‌20 g, *Pinellia ternata* (Thunb.) Makino 10 g, *Citrus aurantium* L. 10 g, *Citrus reticulata* Blanco 20 g, *Poria cocos* (Schw.) Wolf 15 g, *Acorus tatarinowii* Schott 10 g, *Polygala tenuifolia* Willd. 10 g, *Glycyrrhiza uralensis* Fisch. ex DC. 6 g, and *Ziziphus jujuba* Mill. 20 g. The herbs were decocted with water, and administered one dose a day, which was administered 2 times (ie, in the morning and evening).

Dapoxetine (Priligy) was administered at a dosage of 30 mg per session and taken 1-3 h before sexual intercourse.

After 4 weeks of treatment, efficacy was evaluated in patients who had engaged in complete sexual intercourse at least once per week throughout the treatment period.

### Outcome measures

The primary outcome measures included the variation in Intravaginal Ejaculatory Latency Time (IELT) before and after treatment (estimated by the participants), as well as assessments using the Premature Ejaculation Diagnostic Tool (PEDT), Global Rating of Sexual Satisfaction (GRISS), Clinical General Impression Change Scale (CGIC), Traditional Chinese Medicine Quality of Life Evaluation Scale (CQ-11D) and patient-completed effectiveness evaluations conducted pre- and post-treatment.

### Safety evaluation

Patient-reported discomfort and symptoms were documented, including their incidence and severity, along with any adverse effects experienced during the treatment period. The results were subsequently presented through the differential scores obtained from the adverse reaction form.

### Statistical analysis

Statistical analyses were performed using IBM SPSS Statistics for macOS, Version 27.0 (IBM Corp., Armonk, NY, USA). The normality of distribution was evaluated using the Shapiro–Wilk test, while homogeneity of variance was assessed via Levene’s test. Measurement data conforming to a normal distribution with equal variances are presented as mean ± SD (x- ± SD) and were compared between 2 groups using the independent samples *t*-test. Data that did not conform to a normal distribution or exhibited unequal variances are reported as median (interquartile range) [M (P25, P75)] and were analyzed using the Mann–Whitney *U* test. Categorical data are expressed as frequencies and percentages (*n*, %) and were compared using the chi-square test; Fisher’s exact test was applied when the expected frequency in any cell was less than 5. All tests were 2-tailed, and a *P*-value <.05 was considered indicative of statistical significance.

## Results

### General information

**Figure 1:**
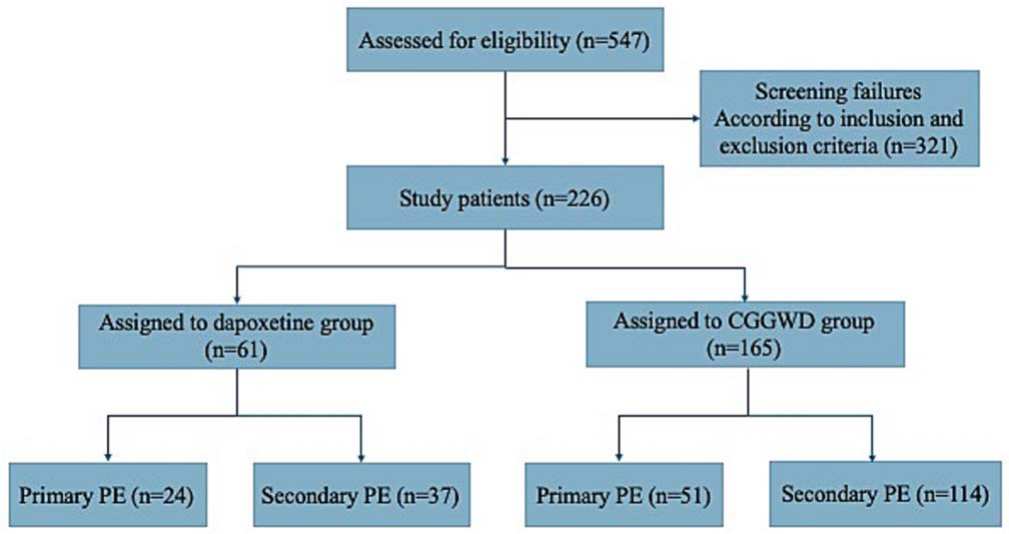
Participant flowchart.

The study comprised a total of 226 participants, with 61 individuals (26.99%) assigned to the dapoxetine group and 165 individuals (73.01%) assigned to the CGGWD group (Figure 1). Statistical analysis revealed no significant differences in age, height, weight, body mass index (BMI), course of the disease, marriage, frequency of sexual intercourse per week and the proportion of primary PE between the 2 groups (All *P* > .05), suggesting that the general demographic and clinical characteristics of the groups were comparable. Statistical analysis showed no significant difference in IELT and PEDT scores between the 2 patient groups before treatment, indicating comparability (all *P* > .05) (Table 1).

**Table 1:** Baseline demographic and clinical characteristics.

**Variable**	**Dapoxetine (*n* = 61)**	**CGGWD (*n* = 165)**	***P*-value**
**Age, year**	30.00 (25.00, 35.00)	31.00 (26.00, 36.00)	.509^c^
**Height, cm**	172.00 (170.00, 177.25)	175.00 (170.00, 178.00)	.090^c^
**Weight, kg**	70.50 (63.00, 79.50)	70.00 (65.00, 79.00)	.588^c^
**BMI, kg/m^2^**	23.62 ± 3.18	23.71 ± 3.04	.845^a^
**Course of the disease, year**	5.00 (2.00, 10.00)	3.00 (1.00, 8.00)	.303^c^
**Married**	62.3%	67.3%	.528^b^
**Frequency of sexual intercourse, time/week**	1.00 (1.00, 2.00)	1 (1.00, 1.50)	.769^c^
**Primary PE**	39.3%	30.9%	.232^b^
**IELT-before, min**	1.50 (1.00, 2.00)	1.50 (1.00, 2.50)	.065^c^
**PEDT-before, score**	13.00 (11.00, 14.00)	12.00 (11.00, 14.00)	.058^c^

Data are presented as mean ± SD, median (IQR) or *n* (%). BMI (body mass index).

^a^ *t*-test. ^b^chi-squared test or Fisher’s exact test. ^c^Mann–Whitney U test.

### Comparison of sexual function changes before and after treatment between the 2 groups

The variations in IELT and PEDT scores pre- and post-treatment in both groups were analyzed (Table 2). In the dapoxetine group, IELT improved from an initial value of 1.50-3.00 min, while in the CGGWD group, it increased from 1.50-3.50 min. The PEDT scores for both groups were compared before and after treatment, with the dapoxetine group’s median dropping from 13 scores to 9 scores and the CGGWD group’s from 12 scores to 9.5 scores. Both groups demonstrated statistically significant improvements in ejaculation latency and PEDT scores following treatment (*P* < .05).

**Table 2:** Comparison among groups of IELT and PEDT changes pre- and post-treatment.

**Variable**	**Dapoxetine (*n* = 61)**	**CGGWD (*n* = 165)**	***P*-value for inter-group comparison**
**IELT, min**			
** Before, M (Q₁, Q₃)**	1.50 (1.00, 2.00)	1.50 (1.00, 2.50)	.065
** After, M (Q₁, Q₃)**	3.00 (1.50, 5.00)	3.50 (2.00, 5.50)	.335
*** P*-value for comparison. With baseline**	<.001	<.001	
**PEDT, score**			
** Before, M (Q₁, Q₃)**	13.00 (11.00, 14.00)	12.00 (11.00, 14.00)	.058
** After, M (Q₁, Q₃)**	9.00 (8.00, 12.00)	9.50 (7.00, 11.00)	.530
***P*-value for comparison. With baseline**	<.001	<.001	
**Comparison of the differences in before and after intervention**			
** IELT-DIF, M (Q₁, Q₃)**	1.50 (0.50, 3.00)	1.50 (0.50, 3.50)	.978
** PEDT-DIF, M (Q₁, Q₃)**	−3.00 (−5.00, −1.00)	−2.00 (−5.00, −1.00)	.567
** Efficiency, M (Q₁, Q₃)**	1.00 (0.50, 2.33)	0.83 (0.20, 2.08)	.325

Data are presented as median (IQR). Group comparisons were performed using the Mann–Whitney *U* test, as the data violated the assumption of normality per the Shapiro–Wilk test.

Efficiency = (post - pre)/pre.

Additionally, changes in IELT and PEDT as well as treatment effectiveness rates before and after treatment were compared between the 2 groups (Table 2). The median increase in IELT was 1.5 min in both the dapoxetine and CGGWD groups. For PEDT, the median decrease was 3 points in the dapoxetine group and 2 points in the CGGWD group. The enhancement in efficiency was assessed using the multiple of IELT as a metric. The dapoxetine group exhibited an increase of 1.0 times, whereas the CGGWD group demonstrated an increase of 0.83 times. However, no statistically significant differences were observed in IELT-DIF, PEDT-DIF, or overall efficacy between the 2 groups (*P* > .05).

### Comparing changes in sexual satisfaction, CQ-11D, and clinical impression pre- and post-treatment

Before treatment, the GRISS satisfaction scores of the 2 groups were not significantly different (*P* > .05). Post-treatment, no significant differences were found in GRISS scores between the 2 groups (*P* > .05). Both groups exhibited significant improvements in sexual satisfaction indicators following treatment, suggesting that both CGGWD and dapoxetine effectively enhance patients’ sexual satisfaction (*P* < .05). Before treatment, the CQ-11D scores of the 2 groups showed no significant difference. Post-treatment, the CGGWD group showed significantly greater improvement than the dapoxetine group (*P* < .05), indicating a more notable enhancement in systemic symptoms. There was no significant difference in CGIC scores between the groups after treatment (*P* > .05), indicating that both groups of patients similarly evaluated the treatment effect (Table 3).

**Table 3:** Comparison among groups of GRISS, CQ11D, and CGIC changes pre- and post-treatment.

**Variables**	**Dapoxetine (*n* = 61)**	**CGGWD (*n* = 165)**	***P*-value for inter-group comparison**
**GRISS, score**			
** Before, M (Q₁, Q₃)**	37.00 (35.00, 42.00)	36.00 (34.00, 40.00)	.065
** After, M (Q₁, Q₃)**	28.00 (24.00, 36.00)	26.50 (21.00, 34.00)	.148
***P*-value for comparison with baseline**	<.001	<.001	
**CQ-11D, score**			
** Before, M (Q₁, Q₃)**	24.00 (22.00, 26.00)	24.00 (22.00, 25.00)	.607
** After, M (Q₁, Q₃)**	24.00 (22.00, 28.00)	19.00 (17.00, 21.00)	<.001
***P*-value for comparison. With baseline**	<.001	<.001	
**Comparison of the differences in before and after intervention**			
** GRISS-DIF, M (Q₁, Q₃)**	−10.00 (−14.00, −5.00)	−10.00 (−15.00, −4.00)	.883
** CQ11D-DIF, M (Q₁, Q₃)**	0.00 (0.00, 2.00)	−4.00 (−6.00, −3.00)	<.001
** CGIC, M (Q₁, Q₃)**	2.00 (1.00, 3.00)	2.00 (1.00, 3.00)	.993

Data are presented as median (IQR). Group comparisons were performed using the Mann–Whitney U test, as the data violated the assumption of normality per the Shapiro–Wilk test.

### Safety assessment

During the treatment process, the common adverse events in the 2 groups included nausea, dizziness, drowsiness, headache, diarrhea, insomnia, erectile dysfunction, and dry mouth (Table 4). Nausea was most frequent in the dapoxetine group, while diarrhea was most common in the CGGWD group. The incidence of adverse reactions was significantly lower in the CGGWD group, with 7 cases (including repeated cases), compared to 29 cases in the dapoxetine group (including repeated cases) (*P* < .05). The dapoxetine group had a higher overall incidence of adverse events compared to the CGGWD group, with a statistically significant difference (*P* < .05). No patients stopped taking the medication due to these adverse events.

**Table 4:** Adverse events during treatment.

**Variables**	**Dapoxetine (*n* = 61)**	**CGGWD (*n* = 165)**	***χ*** ^***2***^	***P*-value**
**Nausea**	9 (14.8%)	0 (0.0%)		
**Dizziness**	5 (8.2%)	0 (0.0%)		
**Drowsiness**	4 (6.6%)	1 (0.6%)		
**Headache**	4 (6.6%)	0 (0.0%)	67.218^a^	<.001^a^
**Diarrhea**	2 (3.3%)	5 (3.0%)		
**Insomnia**	2 (3.3%)	0 (0.0%)		
**Erectile dysfunction**	1 (1.6%)	0 (0.0%)		
**Dry mouth**	2 (3.3%)	1 (0.6%)		

Categorical data are presented as *n* (%).

^a^Fisher’s exact test.

## Discussion

Currently, the management of PE predominantly depends on pharmacological interventions, with SSRIs being the preferred therapeutic agents. Nevertheless, the administration of SSRIs is associated with several limitations, such as diminished sexual desire and erectile dysfunction.^13^ Furthermore, patients may experience “SSRI discontinuation syndrome,”^6^^,^^14^ characterized by a constellation of physical and psychological symptoms, including nausea, vomiting, dizziness, headache, ataxia, drowsiness, agitation, anxiety, and insomnia.^15^ Additionally, emerging research suggests that SSRIs may exert deleterious effects on sperm quality.^16^^,^^17^

Research indicates that TCM has the capacity to restore the balance of serotonin (5-HT) and norepinephrine by inhibiting monoamine oxidase activity and enhancing the reuptake of synaptic neurotransmitters.^18^ Furthermore, TCM demonstrates therapeutic efficacy in patients with PE who also experience anxiety and depression. This is achieved through multi-target interventions that encompass neurotransmitter regulation, metabolic modulation, and neuroendocrine-immune system integration.^19^ Concurrently, TCM exerts regulatory effects on overall physiological functions through complex, multi-target mechanisms, which can alleviate a variety of systemic symptoms.^9^

Current research identifies numerous TCM approaches for addressing PE, including the Qiaoshao formula,^20^ Shugan Yidan Fang,^21^ Ningmitai capsule,^22^ Shuilu Erxian Dan,^23^ and Yimusake tablet.^24^ However, most of these studies have been validated primarily through animal models or treating PE combined with SSRIs, lacking clinical research on using TCM alone.^25^ This study opted to compare dapoxetine treatment for PE with TCM alone.

In this retrospective study, a comparative analysis was conducted between TCM as a standalone treatment and dapoxetine, utilizing multiple validated measures, including IELT and PEDT. In accordance with established guidelines, participants were categorized into 2 groups: a control group receiving the Western medication dapoxetine, and a treatment group administered the TCM Chaihu Guizhi Ganjiang Decoction combined with Wendan Decoction.^26^

Within this study, 26.99% of the participants were treated with dapoxetine, while 73.01% received TCM. Although the disparity in group sizes may introduce selection bias, statistical analyses revealed no significant differences in baseline characteristics between the 2 groups, thereby mitigating concerns regarding this potential bias.

Following a 4-week treatment period, no significant differences were observed between the dapoxetine group and the CGGWD group in terms of IELT, PEDT scores, effective rate, sexual satisfaction as measured by GRISS, and CGIC scores. These findings suggest that CGGWD is effective in the treatment of PE. Furthermore, the CQ-11D indicated that the CGGWD group experienced a significantly better therapeutic effect compared to the dapoxetine group. In the adverse event table, the incidence of adverse events in the CGGWD group was generally lower than that in the dapoxetine group. This suggests that CGGWD is safe and effective in treating PE and can improve patients’ satisfaction with their overall condition.

In TCM, the liver is believed to play a crucial role in regulating emotions. Stagnation of liver *qi* is thought to result in emotional disturbances, potentially influencing neural reflexes through neurotransmitter pathways. Empirical research has demonstrated that liver-soothing and depression-alleviating pharmacological agents can ameliorate conditions such as depression and PE by modulating 5-HT levels.^21^^,^^27^ This study focuses on the Chaihu Guizhi Ganjiang Decoction, a traditional Chinese medicinal compound recognized for its liver-soothing and depression-relieving properties. Notably, it has been reported that the primary constituents of CGGWD can enhance the expression of the dopamine D1 receptor, which may facilitate the regulation of dopaminergic signaling and consequently impact neuronal function in the brain.^28^ Second, Wen Dan Decoction is a traditional formulation renowned for its efficacy in calming the mind. Research indicates that its mechanism in preventing insomnia involves multiple signaling pathways, including neurotransmitter signaling and the NF-κB pathway.^29^ Additionally, it appears to enhance the binding affinity of neurotransmitters to their respective receptors by modulating the transmission levels of amino acids in the brain.^30^ This modulation subsequently regulates several neurotransmitters, such as gamma-aminobutyric acid, 5-HT, and glutamic acid.^31^ However, the precise mechanism of action of CGGWD requires further investigation and validation.

In this retrospective cohort study, the use of CGGWD was associated with comparable improvements in IELT and PEDT when compared to on-demand dapoxetine. Dapoxetine is typically administered as needed prior to sexual intercourse, thus functioning as a symptom-oriented treatment closely aligned with anticipated sexual activity. Conversely, CGGWD in the present study was administered daily. On-demand treatment offers convenience to certain patients by circumventing continuous medication exposure; however, it necessitates premeditated planning prior to sexual activity. Conversely, daily treatment potentially alleviates the necessity for timing medication with intercourse and may be more favorable for patients desiring consistent symptom management. Nonetheless, it imposes a greater daily medication burden and depends on sustained adherence. The superior improvement observed in CQ-11D scores within the CGGWD group may partially be attributed to the holistic approach characteristic of TCM. TCM formulations are typically designed to address comprehensive systemic and individualized symptom patterns in patients with PE.^32^ This conceptual distinction may elucidate the observed superior efficacy of CGGWD in addressing systemic symptoms. Future management strategies for PE could potentially benefit from adopting a more integrative model, wherein diverse therapeutic modalities are selected or combined based on the symptom profile and specific treatment objectives.

This study is subject to several limitations. Firstly, it does not employ a prospective, double-blind, randomized controlled trial design. Due to the observational and retrospective design of this study, participants were not randomly assigned. Specifically, the efficacy assessment included only those patients who engaged in complete sexual intercourse at least once per week for the duration of the treatment period. We acknowledge that this non-randomized approach has inherent limitations and may introduce selection bias. Consequently, the findings of this study may be more representative of a particular subgroup of patients characterized by a higher frequency of sexual activity and greater adherence to treatment protocols. Caution is advised when generalizing these conclusions to the broader population of PE patients who exhibit lower frequencies of sexual activity or suboptimal compliance. Future prospective studies should aim to develop more comprehensive strategies to address issues related to patient attrition and low compliance. Second, the restricted collection of fundamental data may introduce unaccounted variables, potentially leading to information bias; this necessitates further investigation to evaluate potential disparities between the 2 groups. Third, the sole utilization of the 30 mg dose of dapoxetine. The exclusion of the 60 mg dose in our study design may have potentially diminished the observed efficacy within the control group. Fourth, this study did not employ an objective measure, such as the stopwatch intravaginal ejaculatory latency time, to evaluate effectiveness. Furthermore, the absence of subgroup analysis for PE may have influenced baseline characteristics and treatment outcomes. Additionally, employing the IIEF-5 questionnaire to evaluate erectile function constitutes an additional limitation of this study. Despite the IIEF-5’s advantages in terms of brevity and widespread use, it is important to acknowledge a notable limitation: the inclusion of a general satisfaction question may confound the assessment of erectile function in a population with PE, where satisfaction levels are generally low.^33^ Future research should consider employing ED-specific instruments that focus purely on erectile hardness and maintenance. Finally, the study’s failure to incorporate both short-term and long-term follow-up post-treatment represents another limitation.

## Conclusion

In conclusion, this preliminary, retrospective, real-world study contributes novel clinical data pertinent to the treatment of PE. The findings suggest that TCM and herbal remedies are both safe and efficacious, particularly for patients experiencing liver depression and spleen deficiency. Specifically, the study demonstrates that CGGWD is well tolerated within a 4-week treatment period and significantly enhances IELT and other related PE evaluation metrics, including ejaculatory control, psychological well-being, marital satisfaction, and overall symptomatology. These results imply that CGGWD may offer substantial benefits for the comprehensive enhancement of sexual health and physical condition in patients with PE, supporting for the clinical application of CGGWD.

## Supplementary Material

STROBE_checklist_v4_combined_qfag031

## Data Availability

Data will be made available on request.

## References

[1] Porst H, Montorsi F, Rosen RC, Gaynor L, Grupe S, Alexander J. The premature ejaculation prevalence and attitudes (PEPA) survey: prevalence, comorbidities, and professional help-seeking. Eur Urol. 2007;51(3):816–824. 10.1016/j.eururo.2006.07.004 16934919

[2] McCabe MP, Sharlip ID, Lewis R, et al. Incidence and prevalence of sexual dysfunction in women and men: a consensus statement from the fourth international consultation on sexual medicine 2015. J Sex Med. 2016;13(2):144–152. 10.1016/j.jsxm.2015.12.034 26953829

[3] McMahon CG, Lee G, Park JK, Adaikan PG. Premature ejaculation and erectile dysfunction prevalence and attitudes in the Asia-Pacific region. J Sex Med. 2012;9(2):454–465. 10.1111/j.1743-6109.2011.02507.x 22023395

[4] Laumann EO, Rosen. Sexual dysfunction in the United States: prevalence and predictors (vol 281, pg 537, 1999). JAMA. 1999;281(6):1174–1174. 10.1001/jama.281.6.537 10022110

[5] Rosen RC, Althof S. Impact of premature ejaculation: the psychological, quality of life, and sexual relationship consequences. J Sex Med. 2008;5(6):1296–1307. 10.1111/j.1743-6109.2008.00825.x 18422496

[6] Ahn TY, Park JK, Lee SW, et al. Prevalence and risk factors for erectile dysfunction in Korean men: results of an epidemiological study. J Sex Med. 2007;4(5):1269–1276. 10.1111/j.1743-6109.2007.00554.x 17635695

[7] Gul M, Bocu K, Serefoglu EC. Current and emerging treatment options for premature ejaculation. Nat Rev Urol. 2022;19:659–680. 10.1038/s41585-022-00639-5 36008555

[8] Russo A, Capogrosso P, Ventimiglia E, et al. Efficacy and safety of dapoxetine in treatment of premature ejaculation: an evidence-based review. Int J Clin Pract. 2016;70(9):723–733. 10.1111/ijcp.12843 27456527

[9] Li Y, Duan Y, Yu X, et al. Traditional Chinese medicine on treating premature ejaculation: a systematic review and meta-analysis. Medicine (Baltimore). 2019;98(18):e15379. 10.1097/md.0000000000015379 31045785 PMC6504282

[10] Zhang MZ, Chunying, Jin B, et al. Guidelines for the diagnosis and treatment of premature ejaculation with integrative medicine (trial version). Nat J Androl. 2018;24(2):176–181. 10.13263/j.cnki.nja.2018.02.016

[11] Romano L, Arcaniolo D, Spirito L, et al. Comparison of current international guidelines on premature ejaculation: 2024 update. Diagnostics (Basel). 2024;14(16):1819. 10.3390/diagnostics14161819 39202307 PMC11353472

[12] Zheng X, Zheng Y, Zheng Y, Zheng X. Guiding Principles for Clinical Research on New Drug of Traditional Chinese Medicine; China Medical Science and Technology Press, 2002.

[13] Saleh R, Majzoub A, Abu E-HM. An update on the treatment of premature ejaculation: a systematic review. Arab J Urol. 2021;19(3):281–302. 10.1080/2090598x.2021.1943273 34552780 PMC8451625

[14] Horowitz MA, Taylor D. Tapering of SSRI treatment to mitigate withdrawal symptoms. Lancet Psychiatry. 2019;6(6):538–546. 10.1016/s2215-0366(19)30032-x 30850328

[15] Waldinger MD . Pharmacotherapy for premature ejaculation. Curr Opin Psychiatry. 2014;27(6):400–405. 10.1097/yco.0000000000000096 25203721

[16] Koyuncu H, Serefoglu EC, Yencilek E, Atalay H, Akbas NB, Sarica K. Escitalopram treatment for premature ejaculation has a negative effect on semen parameters. Int J Impot Res. 2011;23(6):257–261. 10.1038/ijir.2011.35 21776003

[17] Koyuncu H, Serefoglu EC, Ozdemir AT, Hellstrom WJ. Deleterious effects of selective serotonin reuptake inhibitor treatment on semen parameters in patients with lifelong premature ejaculation. Int J Impot Res. 2012;24(5):171–173. 10.1038/ijir.2012.12 22573230

[18] Feng D-d, Tang T, Lin X-p, et al. Nine traditional Chinese herbal formulas for the treatment of depression: an ethnopharmacology, phytochemistry, and pharmacology review. Neuropsychiatr Dis Treat. 2016;12:2387–2402. 10.2147/ndt.S114560 27703356 PMC5036551

[19] Xie Z, Zhang X, Jia H, Zhang Y. Efficacy of Chinese herbal medicine in the treatment of anxiety and depression in male sexual dysfunction: a systematic review and meta-analysis protocol. Front Psychiatry. 2025;16:1584306. 10.3389/fpsyt.2025.1584306 40386113 PMC12081408

[20] Guo J, Wang F, Zhou Q, et al. Safety and efficacy of traditional Chinese medicine, Qiaoshao formula, combined with dapoxetine in the treatment of premature ejaculation: an open-label, real-life, retrospective multicentre study in Chinese men. Andrologia. 2021;53(1):e13915. 10.1111/and.13915 33236403

[21] Han Q, Guo J, Wang R, et al. Mechanism of Shugan Yidan fan, a Chinese herbal formula, in rat model of premature ejaculation. Basic Clin Androl. 2023;33(1):25. 10.1186/s12610-023-00200-3 37784033 PMC10546682

[22] Longping P, Zhiwei H, Jiaming S, et al. Effect of Ningmitai capsule plus sertraline on patients with premature ejaculation and enlarged seminal vesicles: a randomized clinical trial. J Trad Chin Med (English Edition). 2018;38(2):266–271. 10.1016/j.jtcm.2018.04.001 32186065

[23] Zhang X, Han Z-Y, Nie P, Wang H, Chen J-H, Chen Y. Action mechanism of professor Xu Fusong’s Shuilu Erxian Dan in the treatment of premature ejaculation: an exploration based on network pharmacology and molecular docking technology. Natl J Androl. 2022;28(2):149–156. 10.13263/j.cnki.nja.2022.02.009 37462488

[24] Zhai X, Pang K, Li H, et al. Study on evaluation of toxicology and quality control of Yimusake tablet. J Ethnopharmacol. 2020;263:111443. 10.1016/j.jep.2018.07.016 30012512

[25] Chang X, Xu M, Chen Y, Che C, Du Y, Wang X. Selective serotonin reuptake inhibitors combined with traditional Chinese medicine for premature ejaculation: a systematic review and meta-analysis. Andrology. 2023;11(1):112–124. 10.1111/andr.13307 36193003

[26] Althof SE, Abdo CHN, Dean J, et al. International Society for Sexual Medicine’s guidelines for the diagnosis and treatment of premature ejaculation. J Sex Med. 2010;7(9):2947–2969. 10.1111/j.1743-6109.2010.01975.x 21050394

[27] Wang Y, Huang Y, Zhao M, et al. Zuojin pill improves chronic unpredictable stress-induced depression-like behavior and gastrointestinal dysfunction in mice via the theTPH2/5-HT pathway. Phytomedicine. 2023;120:155067. 10.1016/j.phymed.2023.155067 37716030

[28] Zhu X, Huang Y, Qiu J, et al. Chaihu Guizhi decoction prevents cognitive, memory impairments and sensorimotor gating deficit induced by N-methyl-d-aspartate receptor antibody in mice. J Ethnopharmacol. 2025;337:118806. 10.1016/j.jep.2024.118806 39278296

[29] Dong W-R, Li H, Li Y-F, et al. Mechanism of Huanglian Wendan decoction in improving impaired glucose tolerance based on skeletal muscle NLRP3/caspase-1/IL-1beta, IL-18 pathway. Zhongguo Zhong Yao Za Zhi. 2021;46(17):4480–4487. 10.19540/j.cnki.cjcmm.20210621.401 34581053

[30] Yang C, Cai C, Yang X, et al. Wendan decoction improves learning and memory deficits in a rat model of schizophrenia. Neural Regen Res. 2012;7(15):1132–1137. 10.3969/j.issn.1673-5374.2012.15.002 25722705 PMC4340029

[31] Li L, Wu X, Gong J, et al. Activation of GABA type a receptor is involved in the anti-insomnia effect of Huanglian Wendan decoction. Front Pharmacol. 2024;15:1389768. 10.3389/fphar.2024.1389768 38846089 PMC11153716

[32] Elena C, Fu W, Juo G, Emmanuele JA. Effects and prospects of the integration of traditional Chinese medicine with Western biomedical approach for premature ejaculation. Integr Med Nephrol Androl. 2022;9(1):7. 10.4103/2773-0387.345766

[33] Xi Y, Colonnello E, Ma G, et al. Validity of erectile function assessment questionnaires in premature ejaculation patients: a comparative study between the abridged forms of the international index of erectile function and proposal for optimal Cutoff redefinition. J Sex Med. 2021;18(3):440–447. 10.1016/j.jsxm.2020.11.018 33384239

